# Monthly Record of Deaths in the City of Buffalo

**Published:** 1857-06

**Authors:** C. L. Dayton

**Affiliations:** Health Physician


ART. IV.— *Report of Mortality in Buffalo for the month of April,* 1857. By C. L. DAYTON, M. D., Health Physician.

| DISEASES. | No. | Males. | Females. |
|---|---|---|---|
| Aneurism, | 2 | 1 | 1 |
| Accident, | 1 | 1 | |
| Asphyxia Immersorum, | 3 | 2 | 1 |
| Abscess, Cystic, with Fractured Femur, | 1 | 1 | |
| Croup, | 5 | 3 | 2 |
| Cholera Morbus, | 1 | 1 | |
| Cephalitis, | 4 | 2 | 2 |
| Cancer of the stomach, | 2 | | 2 |
| Dysentery | 2 | 1 | 1 |
| Diarrhœa, | 2 | | 2 |
| Dentitio, | 1 | 1 | |
| Debilitas Senilis, | 5 | 2 | 3 |
| Enteritis, | 2 | 1 | 1 |
| Eclampsia, | 20 | 16 | 4 |
| Febris Typhoid, | 3 | 3 | |
| " Scarlatina sequelæ of, | 26 | 13 | 13 |
| Hyperæmia Cerebralis, | 2 | 1 | 1 |
| " Pulmonalis, | 5 | 2 | 3 |
| " Puerperarum, | 1 | | 1 |
| Hydrocephalus, | 8 | 5 | 3 |
| Hydrops, | 1 | 1 | |
| Icterus, | 1 | | |
| Ignotus, | 11 | 7 | 4 |
| Intemperance, | 2 | | 2 |
| Laryngitis et Tracheitis, | 1 | | 1 |
| Marasmus, | 9 | 5 | 3 |
| Morbus Hepatis, | 1 | | 1 |
| " Cordis, | 3 | 2 | 1 |
| Pertussis, | 1 | | 1 |
| Parturitio, | 1 | | 1 |
| Pneumonitis, | 12 | 4 | 6 |
| Peritonitis Puerperal, | 1 | | 1 |
| Paralysis, occasioned by Lead, | 1 | 1 | |
| Rheumatism, | 2 | 1 | 1 |
| Still-born, | 6 | 4 | 1 |
| Scrophula, | 1 | | 1 |
| Thrush, | 1 | | 1 |
| Tumor, | 1 | 1 | |
| Tuberculosis Pulmonalis, | 26 | 11 | 15 |
| Total, | 173 | | |

SEXES.

| | |
|---|---|
| Males, | 83 |
| Females, | 80 |
| Sex not given, | 5 |
| Total, | 178 |

AGES.

| | | | |
|---|---|---|---|
| Still-born, | 6 | Between 20 years and 30 years, | 13 |
| 1 day, | 2 | " 30 " " 40 " | 14 |
| 1 day and 30 days, | 9 | " 40 " " 50 " | 9 |
| Between 1 month and 6 months, | 20 | " 50 " " 60 " | 6 |
| " 6 months and 12 " | 19 | " 60 " " 70 " | 5 |
| " 1 year " 3 years, | 33 | " 70 " " 80 " | 2 |
| " 3 " " 5 " | 13 | " 80 " " 90 " | 1 |
| " 5 " " 10 " | 16 | " 90 " " 100 " | 0 |
| " 10 " " 20 " | 9 | " 100 " | 1 |
| Total number under 10 years, | 118 | Total number over 10 years, | 60 |

Ages not given, 0 Total, 178

NATIVITIES.

| | | | |
|---|---|---|---|
| American, (white,) | 88 | Dane, | 1 |
| " (colored,) | 3 | French, | 1 |
| German, | 36 | Scotch, | 2 |
| Irish, | 16 | Swede, | 1 |
| English, | 2 | Country not given, | 28 |
| Canadian, | 0 | | |

Total, 178

---

*Solution of Phosphoric Acid in Typhus.* By Professor MAGNUS HUSS of Stockholm.

Solution of phosphoric acid, two and a quarter ounces; decoction of marsh mallow, five ounces; syrup of marsh mallow, four ounces; mix—dose, from ten to fifteen drops every two hours. M. Huss recommends this solution in the first stage of typhus, whether it appear under the abdominal or petechial form, or under any form intermediate between these. The state of the tongue by no means contraindicates the use of this remedy, which always renders the course of the disease more favorable. The solution contains 25 per cent. of phosphoric acid.—*Presse Médicale Belge.—Dublin Medical Press.*

